# BOAT: Basic Oligonucleotide Alignment Tool

**DOI:** 10.1186/1471-2164-10-S3-S2

**Published:** 2009-12-03

**Authors:** Shu-Qi Zhao, Jun Wang, Li Zhang, Jiong-Tang Li, Xiaocheng Gu, Ge Gao, Liping Wei

**Affiliations:** 1 Center for Bioinformatics, National Laboratory of Protein Engineering and Plant Genetic Engineering, College of Life Sciences, Peking University, Beijing 100871, PR China

## Abstract

**Background:**

Next-generation DNA sequencing technologies generate tens of millions of sequencing reads in one run. These technologies are now widely used in biology research such as in genome-wide identification of polymorphisms, transcription factor binding sites, methylation states, and transcript expression profiles. Mapping the sequencing reads to reference genomes efficiently and effectively is one of the most critical analysis tasks. Although several tools have been developed, their performance suffers when both multiple substitutions and insertions/deletions (indels) occur together.

**Results:**

We report a new algorithm, Basic Oligonucleotide Alignment Tool (BOAT) that can accurately and efficiently map sequencing reads back to the reference genome. BOAT can handle several substitutions and indels simultaneously, a useful feature for identifying SNPs and other genomic structural variations in functional genomic studies. For better handling of low-quality reads, BOAT supports a "3'-end Trimming Mode" to build local optimized alignment for sequencing reads, further improving sensitivity. BOAT calculates an E-value for each hit as a quality assessment and provides customizable post-mapping filters for further mapping quality control.

**Conclusion:**

Evaluations on both real and simulation datasets suggest that BOAT is capable of mapping large volumes of short reads to reference sequences with better sensitivity and lower memory requirement than other currently existing algorithms. The source code and pre-compiled binary packages of BOAT are publicly available for download at http://boat.cbi.pku.edu.cn under GNU Public License (GPL). BOAT can be a useful new tool for functional genomics studies.

## Background

Next generation sequencing technologies have been widely used in biology research, such as in genome-wide identification of polymorphisms, transcription factor binding sites, methylation states, and transcript expression profiles [1]. With these ultra high-throughput sequencing technologies, massive amounts of short sequencing reads can be generated rapidly at low cost. For example, the Solexa system from Illumina can generate 30 M reads and 1 G bases (single end) or 2 G bases (pair-end) in a single run [2]. The large volume of data poses serious challenges for effective data analysis.

One of the most critical analysis tasks is to map the sequencing reads to reference sequences accurately and efficiently. General alignment tools such as BLAST [3] and BLAT [4] suffer from long running time. New dedicated algorithms such as ELAND (unpublished), SOAP [5], MAQ [6], RMAP [7] and SeqMap [8] have been developed to achieve better mapping efficiency. Among these algorithms, ELAND, MAQ and SOAP employ similar seed index and search schema, except that ELAND and MAQ create index for query reads and SOAP creates index for reference sequences. ELAND can handle up to 2 substitutions, while MAQ can handle up to 3 substitutions. RMAP is mainly developed for handling mutations in 3' low quality region, but it lacks the sensitivity for leading sequence mutations. While these algorithms are effective in handling near-perfect matches, their mapping sensitivity, speed, and/or memory requirement suffer when handling simultaneous multiple substitutions and indels.

While many attempts have been made to improve sequencing accuracy, the next-generation sequencing platforms still suffer from significantly higher error rate when being compared to classical Sanger sequencing. Statistics on the number of wrong base calls at each base position of typical Solexa reads showed that the sequencing error rates range from 0.3% at the beginning of reads to 3.8% near the end of reads, and may reach up to 11.8% at the last base [9]. Moreover, recent studies have revealed that genome variations like SNPs and small-scale indels are common in populations and play key roles in diseases as well as individual differences [10,11]. For example, in one of the extreme known cases, sequencing of *Ciona savignyi *in a natural population revealed a SNP heterozygosity of 4.5% and average per-base indel heterozygosity of 16.6% [12]. In human, somatic point mutation rates were found to be 1000 times higher in 13% of sporadic colorectal cancers infected by MIN (microsatellite instability) tumors than in normal cells [11].

Thus, there is a need for a new mapping algorithm that can effectively handle simultaneous multiple substitutions and indels. Here we present such a new algorithm, Basic Oligonucleotide Alignment Tool (BOAT). Evaluations on both real and simulation datasets revealed that BOAT has better performance than other existing tools.

## Results and discussion

BOAT can handle several substitutions and indels simultaneously using adaptive indexing and searching strategies (see Methods and materials). It is optimized for mapping single-end and paired-end Solexa reads to a reference genome, but can also map SAGE, MPSS and 454 reads. BOAT does not require that all reads have the same length. It calculates an E-value for each hit as mapping quality assessment and provides customizable post-mapping filters for further mapping quality control. BOAT can be run on most UNIX-like platforms such as Linux and Solaris as a standard Unix/Linux command line program. It supports multiple threads scheduling and can use CPU resources effectively on both desktop PCs and large-scale computer farm. Both the source code and pre-compiled binary packages of BOAT are available for free download at http://boat.cbi.pku.edu.cn under GNU Public License (GPL).

To evaluate and compare the performance of BOAT, we first mapped 8,755,069 Solexa reads generated in RNA-sequence experiments [13] back to the mouse genome (mm9 assembly) using BOAT (v1.0) and four other existing programs, MAQ (v0.6.8), RMAP (v0.41), SeqMap (v1.0.8) and SOAP (v1.11). Since all of these four programs support three mismatches within 33-mer full read length, we allowed up to three mismatches, including substitutions and indels, during the mapping. As shown in Table 1, BOAT achieved the highest sensitivity at less memory requirement and less or comparable time cost. For example BOAT used only 65% of execution time (9,621 min vs. 14,654 min) and mapped 3.5% more reads (4,713,133 vs. 4,555,705) compared to the second most sensitive program SOAP.

**Table 1:** Performance comparison based on a real dataset.

	Number of mapped reads	Time(min)	Memory(MB)
BOAT	4,713,133	9,621	1,415
SOAP	4,555,705	14,654	1,215
RMAP	4,520,282	34,774	3,448
SeqMap	4,339,235	18,593	20,529
MAQ	3,879,236	1,127	2,897

8,755,069 RNA-seq profiling Solexa reads were mapped to mouse whole genome with different programs. In this comparison, the maximum mismatch number threshold was set to 3 (including substitutions and indels). The comparison was run on a local Linux box with two Intel quad-core (E7310 @ 1.6 G Hz) CPUs and 64 G RAM (detailed running parameters for each tool were shown in Supplementary Table S1 of Additional File 2). To handle the physical memory limitation of some of the programs BOAT is compared to, reads were mapped against individual chromosomes sequentially. "Time" shows the sum of the execution times, and "Memory" shows the maximal memory usage among those runs.

Further comparison on simulation data revealed more advantage of BOAT over other programs. Here we used the same module proposed by MAQ [6] to generate five million 33-mer simulated reads with 100,279 mutations (the substitution rate was about 5% and the indel rate was about 1.5%) from a two-million-bp region on mouse chromosome X. The simulated reads were then mapped back to the X chromosome. As shown in Table 2, BOAT achieved higher sensitivity (76.56%) and precision (99.41%) compared to other tools, having mapped about 30% more reads than the second best algorithm RMAP, with moderate memory and time cost. As shown in Supplementary Figure S1 (Additional File 1) the increase in sensitivity was especially prominent when the number of mismatches was high.

**Table 2:** Performance comparison based on a simulation dataset.

	Number of mapped reads	Recall	Precision	Time(min)	Memory(MB)
BOAT	3,833,479	76.56%	99.41%	18	1,217
RMAP	2,957,658	58.89%	98.90%	840	2,371
SOAP	2,872,535	56.75%	97.19%	9	186**
MAQ	2,878,570	55.93%	93.53%	4	1,959
SeqMap*	2,187,611	43.57%	99.25%	33	12,500

5,000,000 simulated reads were mapped to an original two-million-bp mouse chrX region on a local Linux box with two Intel quad-core (E7310 @ 1.6 G Hz) CPUs and 64 G RAM. All programs were tuned to maximize their capability for tolerating no more than five mismatches (detailed running parameters for each tool were shown in Supplementary Table S2 of Additional File 2).

* We tried to run SeqMap with up to 5 mismatches, but failed with out-of-memory error. So only 3 mismatches with 1 indel were allowed when running SeqMap.

** As only a small part of the whole genome was used as reference sequence in this benchmark, the memory usage of SOAP is very low. However, when mapping to the whole human genome, at least 14 GB memory is required to run SOAP [5].

BOAT provides flexible and friendly features. A comparison of its features against other tools is shown in Table 3. In addition to the default mode, BOAT supports a "Quick Mode" dedicated to identify nearly perfect match, achieving over 10-fold speed-up at the cost of ignoring hits with more than one mismatch. On the other hand, to better handle low-quality reads, BOAT supports a "3'-end Trimming Mode" to construct best local alignment instead of optimizing for the global alignment between the reads and the reference sequences. This is useful in dealing with sequencing reads with low-quality tail region or small RNA analysis with adaptor included in the tail region. BOAT also provides an auxiliary program *SNPcall *to identify SNP sites based on mapped reads. To reduce potential false positives caused by sequencing errors, *SNPcall *masks sites with low quality scores before performing SNP calling. By applying *SNPcall *with default criteria (at least four supporting reads *per site*) to the simulated dataset, 80.89% (81,111 out of 100,279) true SNPs were recovered, a much higher recovery rate than that from MAQ's SNP discovery pipeline (54.69%) (Supplementary Figure S2 in Additional File 1). This could be partly attributed to the fact that MAQ did not support identification of SNPs around indels in single-end reads [6].

**Table 3:** Feature comparison of BOAT and other commonly used Solexa read mapping programs

	Maximum number of mismatches allowed	Gapped alignment	Trimming alignment	BLAST-style E-value	Pair-end reads	SNP Calling
BOAT	No hardcoded limitation	YES	YES	YES	YES	YES
RMAP	No hardcoded limitation	NO	NO	NO	NO	NO
MAQ	3	NO	NO	NO	YES	YES
SOAP	5	NO	YES*	NO	YES	NO
SeqMap	5	YES	NO	NO	NO	NO

* SOAP provided a similar mode called "iterative alignment" by iteratively trimming base pairs at the 3'-end and redoing the alignment until hits are detected or the remaining sequence is too short.

## Conclusion

Benchmark based on both real and simulation datasets suggested that BOAT offered better sensitivity with lower memory requirement and comparable or lower time cost than other existing tools. Effectively handling multiple substitutions and indels simultaneously could make full use of sequencing data. BOAT could be a valuable tool in functional genomic studies.

## Methods

**Figure 1:**
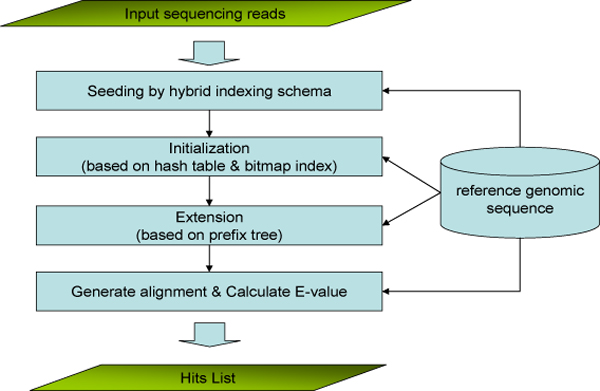
**Flow chart of the BOAT algorithm**. BOAT takes the leading sequence of a read as seed to initialize an alignment and extends the alignment by traversing through the prefix tree that stores the sequence of the read.

BOAT takes as input a reference genomic sequence and a set of sequencing reads from Solexa, 454, SAGE or MPSS. The flow chart of BOAT is shown in Figure 1 and the algorithm is described below.

### Query seed index

To effectively handle the large data volume generated by the new ultra high-throughput sequencing technologies, BOAT builds index for query reads instead of the reference sequence. To handle multiple mixed substitutions and indels, BOAT employs a hybrid indexing schema, combining hash table, bitmap index and prefix tree for better performance (Supplementary Figure S3 in Additional File 1). Since the sequencing quality at the 5' end is much better than that at the 3' end [9], BOAT creates an index and initializes an alignment based on the leading fragment of a sequencing read. It uses two *n*-mer discontinuous fragments separated by *m*-mer gap as seeds for each read. These seeds are pre-indexed as hash tables for fast searching and the gap between the seeds is used as bitmap index. To further speed up alignment search, BOAT organizes the sequencing reads in a prefix tree and records the entrance of tree in hash table for each seed. Up to thirteen bases are compressed into each prefix tree node to reduce memory requirement. Such a hybrid schema provides linear time searching (g gaps in *O*(gn) and k substitutions in *O*(kn)) with efficient memory usage.

### Mapping reads against reference sequence

The mapping process involves two steps: (A) alignment initialization with indexed seeds: the alignment search will be initialized only when either of the two indexed seeds contains no more than one mismatch. (B) Alignment extension with prefix tree: BOAT extends the initialized alignment by performing depth-first search within the pre-indexed prefix tree. The search will backtrack to the most recent un-visited node after a) exceeding the mismatch number tolerance or b) reaching the leaf nodes.

If only nearly-perfect matches are expected, a "Quick Mode" search schema can be used which triggers alignment extension only when perfect match detected for at least one seed, which further improves performance by one order of magnitude. On the other hand, when large differences are expected between the reads and reference sequences, it may not be possible to build a global alignment with the full length of reads covered. To handle these cases, BOAT provides a "3'-end Trimming Mode" to construct best local alignment instead. Here, BOAT records the best local alignment location for each read when applying the depth-first search and reports them if no global full-length alignment could be made under the given mismatch tolerances.

### Measuring mapping quality

#### E-value and bit score

To assess alignment quality, BOAT derives a BLAST-style E-value and the corresponding bit score for each hit based on Karlin-Altschul statistics[18]. For increased sensitivity a loose scoring schema (+1, -1 for match and mismatch and -2, -1 for gap opening and extension penalty) is used as suggested by literatures [18,19]. To avoid the potential bias caused by short fragments, BOAT calculates the E-value based on the whole query read. This results in a more accurate estimation of the alignment quality.

#### Evaluation criteria on the simulation benchmark dataset

Because it is difficult to estimate the Specificity in the sequence mapping context, partly due to the difficulty in assessing True Negative (i.e. the number of unmatched reads that are not derive from the reference sequence) [6,7], we instead used Recall and Precision to measure the performance of different tools:

where *TP *(*True Positive*) is the number of reads that are correctly mapped to its original locus, *FP *(*False Positive*) is the number of reads that are not mapped to their original locus, and *FN *(*False Negative*) is the number of reads that failed to be mapped to the reference.

## Competing interests

The authors declare that they have no competing interests.

## Authors' contributions

SQZ, LZ, GG and LW conceived the research; SQZ and JW wrote the code; SQZ, LZ, JTL, GG, and LW analyzed the data; SQZ and GG wrote the first draft of the manuscript, JW, LZ, JTL, XCG, GG and LW revised the manuscript. All authors have read and approved the final manuscript.

## Note

Other papers from the meeting have been published as part of *BMC Bioinformatics *Volume 10 Supplement 15, 2009: Eighth International Conference on Bioinformatics (InCoB2009): Bioinformatics, available online at http://www.biomedcentral.com/1471-2105/10?issue=S15.

## Supplementary Material

Additional file 1 **The detailed analysis of benchmark result and the sequencing reads index schema of BOAT**. Supplementary Figure S1 contains the number of mapped reads classified by the mismatch number for simulation dataset. Supplementary Figure S2 contains assessment of Sensitivity and Precision of SNP discovery by BOAT SNPcall function. Supplementary Figure S3 demonstrates the sequencing reads index schema of BOAT. Click here for file

Additional file 2 **running parameters of all programs in benchmark comparison**. Supplementary Table S1 shows the running parameters of all programs for real dataset. Supplementary Table S2 shows the running parameters of all programs for simulation dataset. Click here for file
