# Supplementary material for: BOAT: Basic Oligonucleotide Alignment Tool
Source: BMC Genomics. 2009 Dec 3;10(Suppl 3):S2. doi: 10.1186/1471-2164-10-S3-S2 (PMC2788372; doi:10.1186/1471-2164-10-S3-S2)

Additional file 1 - The detailed analysis of benchmark result and the sequencing reads index schema of BOAT

Supplementary Figure S1 contains the number of mapped reads classified by the mismatch number for simulation dataset. Supplementary Figure S2 contains assessment of Sensitivity and Precision of SNP discovery by BOAT *SNPcall* function. Supplementary Figure S3 demonstrates the sequencing reads index schema of BOAT.

Supplementary Figure S1 – the number of mapped reads classified by the mismatch number for simulation dataset

*We tried to run SeqMap with up to 5 mismatches, but failed with out-of-memory error on the 64G RAM test box. So only 3 mismatches (with 1 indel) were allowed when running SeqMap.

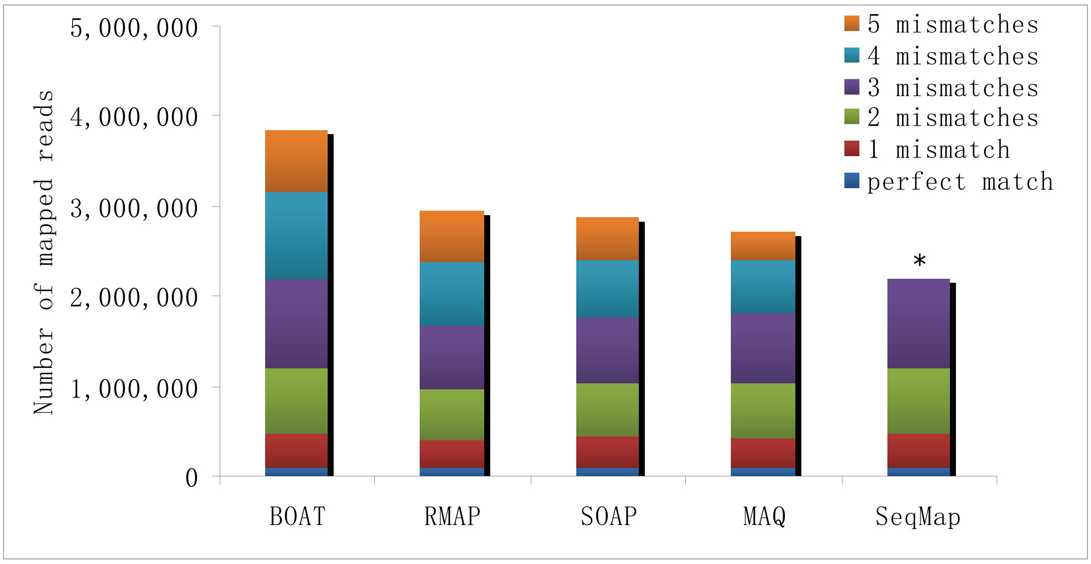

Supplementary Figure 2 - Assessment of Sensitivity and Precision of SNP discovery by BOAT *SNPcall* function

The sensitivity and precision of SNP discovery by BOAT's *SNPcall* function were assessed for the minimum supporting read number ranging from 2 to 20 (marked beside corresponding points). BOAT's *SNPcall* function showed clearly better performance than that of MAQ (highlighted as orange diamond): 47.9% sensitivity improvement for the same precision (highlighted as red square) and 16.3% precision improvement for the same sensitivity (highlighted as green circle).

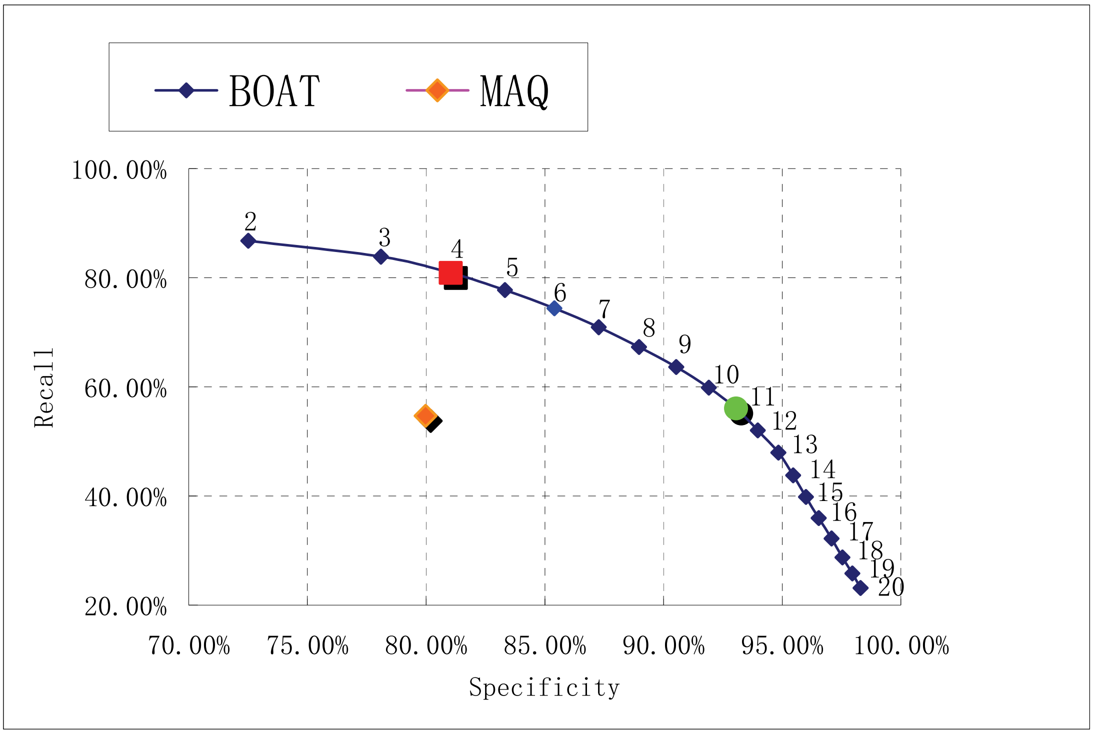

Supplementary Figure 3 - sequencing reads index schema

BOAT takes a hybrid indexing schema to build index for query reads. The hash table, bitmap index and prefix tree are combined together to improve the performance.

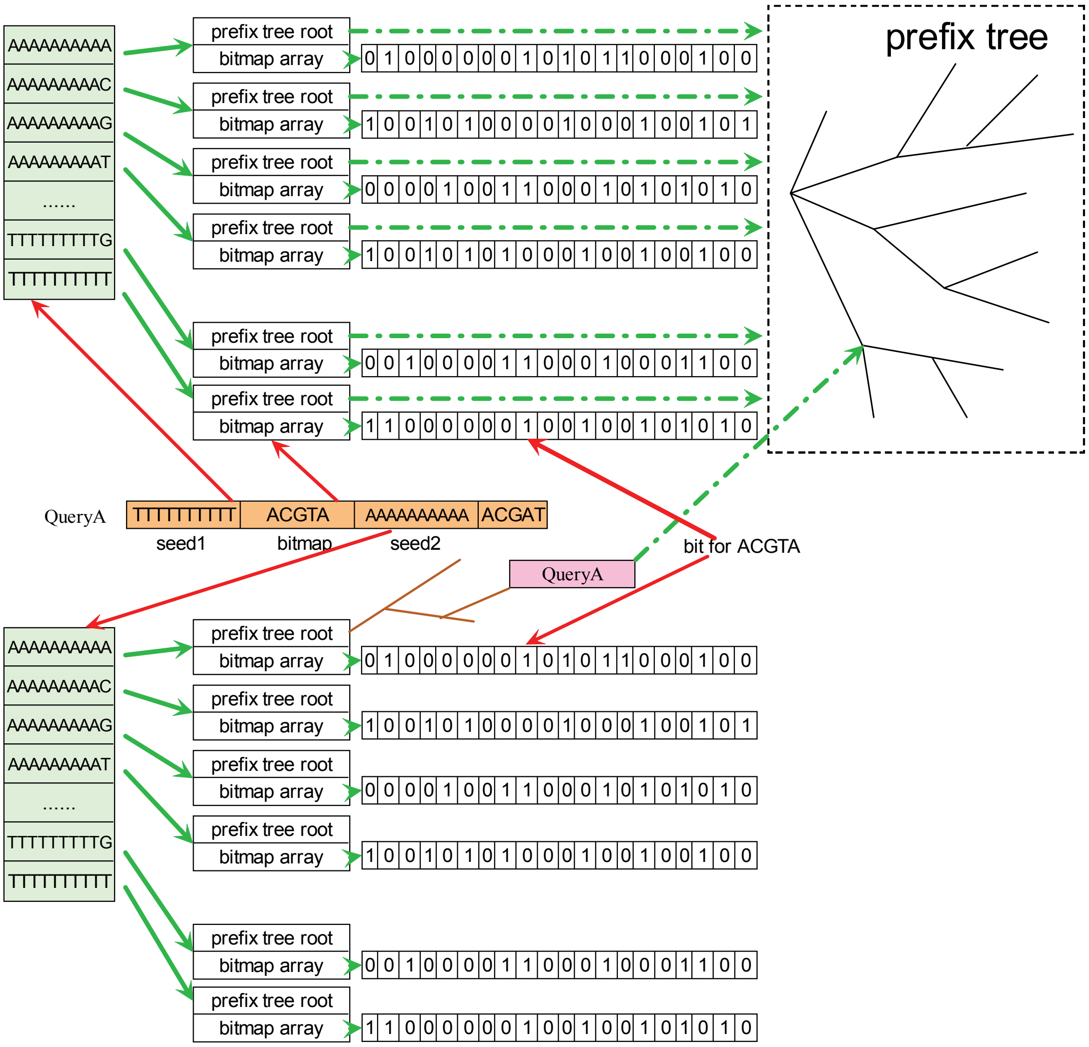

Supplement: Additional file 1 — The detailed analysis of benchmark result and the sequencing reads index schema of BOAT. Supplementary Figure S1 contains the number of mapped reads classified by the mismatch number for simulation dataset. Supplementary Figure S2 contains assessment of Sensitivity and Precision of SNP discovery by BOAT SNPcall function. Supplementary Figure S3 demonstrates the sequencing reads index schema of BOAT. [file 1471-2164-10-S3-S2-S1.pdf]
