# Supplementary material for: BOAT: Basic Oligonucleotide Alignment Tool
Source: BMC Genomics. 2009 Dec 3;10(Suppl 3):S2. doi: 10.1186/1471-2164-10-S3-S2 (PMC2788372; doi:10.1186/1471-2164-10-S3-S2)
Supplement: Additional file 2 — running parameters of all programs in benchmark comparison. Supplementary Table S1 shows the running parameters of all programs for real dataset. Supplementary Table S2 shows the running parameters of all programs for simulation dataset. [file 1471-2164-10-S3-S2-S2.pdf]

Additional file 2 - running parameters of all programs in benchmark comparison

Supplementary Table S1 shows the running parameters of all programs for real dataset.

Supplementary Table S2 shows the running parameters of all programs for simulation dataset.

Supplementary Table S1 – the running parameters of all programs for real dataset comparison

Program	Parameter set
BOAT	-n 3 –g
SOAP	-v 3 -g 3 -r 2
RMAP	-w 33 -m 3
SeqMap	3 /allow_insdel:1 /output_all_matches /do_not_output_probe_without_match
MAQ	-n 3

Supplementary Table S2 –the running parameters of all programs for simulation dataset comparison

Program	Parameter set
BOAT	-n 5 -g -e 1 -r 0
SOAP	-v 5 -g 5 -r 2
RMAP	-w 33 -m 5
SeqMap	3 /allow_insdel:1 /output_all_matches /do_not_output_probe_without_match
MAQ	-n 3
